# Mouse *Rad1 *deletion enhances susceptibility for skin tumor development

**DOI:** 10.1186/1476-4598-9-67

**Published:** 2010-03-24

**Authors:** Lu Han, Zhishang Hu, Yuheng Liu, Xiangyuan Wang, Kevin M Hopkins, Howard B Lieberman, Haiying Hang

**Affiliations:** 1 National Laboratory of Biomacromolecules, Institute of Biophysics, Chinese Academy of Sciences, 15 Road Datun, Beijing 100101, China; 2 Center for Computational and Systems Biology, Institute of Biophysics, Chinese Academy of Sciences, Beijing 100101, China; 3 Department of Genetics & Development, Columbia University Medical Center, 1130 St Nicholas Avenue, Room 311B, New York, NY 10032, USA; 4 Obstetrics & Gynecology, Columbia University Medical Center, 1130 St Nicholas Avenue, Room 311B, New York, NY 10032, USA; 5 The Institute for Human Nutrition, Columbia University Medical Center, 1130 St Nicholas Avenue, Room 311B, New York, NY 10032, USA; 6 Herbert Irving Comprehensive Cancer Center, Columbia University Medical Center, 1130 St Nicholas Avenue, Room 311B, New York, NY 10032, USA; 7 Center for Radiological Research, Columbia University, College of Physicians and Surgeons, 630 W 168thSt, New York, NY 10032, USA; 8 Department of Environmental Health Sciences, Columbia University, Mailman School of Public Health, 60 Haven Avenue, Suite B1, New York, NY 10032, USA

## Abstract

**Background:**

Cells are constantly exposed to stresses from cellular metabolites as well as environmental genotoxins. DNA damage caused by these genotoxins can be efficiently fixed by DNA repair in cooperation with cell cycle checkpoints. Unrepaired DNA lesions can lead to cell death, gene mutation and cancer. The Rad1 protein, evolutionarily conserved from yeast to humans, exists in cells as monomer as well as a component in the 9-1-1 protein complex. Rad1 plays crucial roles in DNA repair and cell cycle checkpoint control, but its contribution to carcinogenesis is unknown.

**Results:**

To address this question, we constructed mice with a deletion of *Mrad1*. Matings between heterozygous *Mrad1 *mutant mice produced *Mrad1*^+/+ ^and *Mrad1*^+/- ^but no *Mrad1*^-/- ^progeny, suggesting the *Mrad1 *null is embryonic lethal. *Mrad1*^+/- ^mice demonstrated no overt abnormalities up to one and half years of age. DMBA-TPA combinational treatment was used to induce tumors on mouse skin. Tumors were larger, more numerous, and appeared earlier on the skin of *Mrad1*^+/- ^mice compared to *Mrad1*^+/+ ^animals. Keratinocytes isolated from *Mrad1*^+/- ^mice had significantly more spontaneous DNA double strand breaks, proliferated slower and had slightly enhanced spontaneous apoptosis than *Mrad1*^+/+ ^control cells.

**Conclusion:**

These data suggest that *Mrad1 *is important for preventing tumor development, probably through maintaining genomic integrity. The effects of heterozygous deletion of *Mrad1 *on proliferation and apoptosis of keratinocytes is different from those resulted from *Mrad9 *heterozygous deletion (from our previous study), suggesting that *Mrad1 *also functions independent of *Mrad9 *besides its role in the Mrad9-Mrad1-Mhus1 complex in mouse cells.

## Background

Living organisms are continuously exposed to both physiological and environmental DNA-damaging agents. Eukaryotic cells have developed exquisite mechanisms that monitor and coordinate cell cycle progression with repair of DNA damage to maintain genome integrity. Mutations in genes that play roles in cell cycle checkpoint control and DNA repair are often associated with tumorigenesis [1,2]. *Rad9, Rad1 *and *Hus1 *are a group of genes conserved from yeast to human that play key roles in cell cycle checkpoints and DNA repair [3-8]. Their protein products form a heterotrimeric ring-like complex, called 9-1-1 [9-11]. It is believed that this complex is important for the function of these three proteins in DNA repair as well as activation of cell cycle checkpoints. It is not clear whether Rad1, Rad9 and Hus1 also have distinct functional activities independent of the heterotrimeric form. The *S. cerevisiae *checkpoint protein Rad17, the orthologue of human Rad1, forms a homocomplex in response to treatment with DNA damaging agents, and the complex is required for yeast survival after exposure to genotoxic agents [12]. Besides the existence of 9-1-1 heterotrimer in K562 and 293 human cells, a significant amount of hRad1 also exists in monomeric form, but monomeric hRad9 and hHus1 were not detectable in a study by Karnitz's group [10] and in our unpublished experiments in 293 human cells. These data suggest a possibility that Rad1 in humans and mice might have distinct functions independent of the 9-1-1 heterotrimer.

Increased expression of Rad9 was found in lung, breast and prostate tumors, relative to normal corresponding tissues [13-16]. High level of Hus1 expression correlates with poor prognosis for ovarian tumors [17]. Knockdown of Rad9 in prostate tumor cells correlates with reduction of tumorigenicity in nude mice [16]. Rad9 knockdown also suppresses growth of human lung adenocarcinoma cells A549 and PC3 [18]. It is likely that increased Rad9 expression is needed for proliferation of tumor cells by mechanisms such as getting beyond (tolerating) oncogene-induced replicative stress and enhancing DNA repair capability. However, mice with conditional deletion of Rad9 in skin keratinocytes are inherently susceptible to the development of skin tumors in response to treatment with the carcinogen 7,12-dimethylbenzanthracene (DMBA)[19]. Thus far, there has been no report addressing the function of Rad1 in carcinogenesis.

To determine whether Rad1 functions to maintain genomic stability and prevent tumor development, we generated *Mrad1 *mutant mice by gene targeting. Homozygous deletion of *Mrad1 *leads to embryonic lethality, but heterozygous animals have no overt defects compared to *Mrad1*^+/+ ^mice. Combined treatment with DMBA and TPA induced skin tumors significantly more frequently in *Mrad1*^+/- ^mice than in *Mrad1*^+/+ ^controls, and also caused significantly more and larger skin tumors in the mutant. *Mrad1*^+/- ^keratinocytes contained more double strand DNA breaks as well, suggesting that this gene is critical for genome stabilization in keratinocytes, and that it carries a function important for preventing tumor development.

## Results

### Mouse embryonic lethality caused by *Mrad1 *homozygous deletion

**Figure 1:**
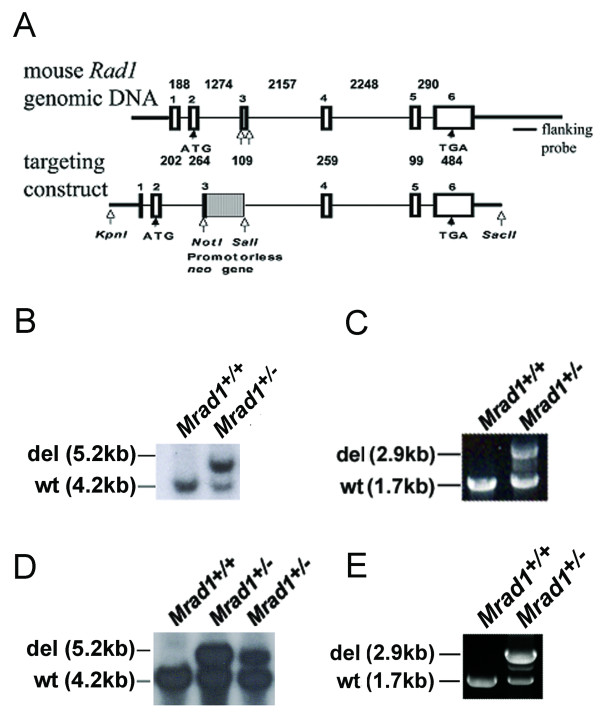
**Targeted deletion of *Mrad1***. *A*, Top panel: *Mrad1 *genomic DNA; Bottom panel: targeting construct. White boxes: exons; Gray box: promotorless *neo *gene; black thin lines: introns; Black thick lines: DNA sequences outside of *Mrad1 *genomic DNA; Numbers above: lengths of introns in bp; Numbers below: lengths of exons in bp; White arrows: restriction enzyme cutting sites (targeting construct) and locations around the DNA sequence to be removed (genomic DNA); *B*, Southern blot analysis of the *Mrad1 *gene in mouse ES cells. Bands indicate wild-type and deleted *Mrad1 *allele. *C*, PCR to assess genotypes in mouse ES cells. Bands indicate wild-type and deleted *Mrad1 *allele. *D*, Southern blot analysis of the *Mrad1 *gene in mice. *E*, *Mrad1 *genotyping in mice using PCR.

To obtain *Mrad1*-disrupted ES cells, we used a promoterless gene targeting strategy to delete the gene [20]. *Mrad1 *was disrupted by homologous recombination in ES cells using the targeting vector illustrated in Fig. 1A. This targeting construct contains a selectable *neo *gene. This gene, which lacked the start codon (ATG), was inserted between the third exon and third intron of *Mrad1*, deleting parts of the third exon and third intron. Homologous recombination of the targeting construct into mouse genomic *Mrad1 *was predicted to generate mutant cells that express a fusion protein containing the 77AA of the Mrad1 N-terminus and a full length neo protein, but lacking the rest of the Mrad1 protein (part of the third exon and the complete 4, 5 and 6 exons). We then targeted a 129Sv/Ev ES cell line obtained from Dr. Victor Lin's laboratory (Columbia University) and *Mrad1*^+/- ^ES cell clones were obtained after transfection and challenge with 300 μg/ml G418. Multiple heterozygous Mrad1-deleted clones were identified by Southern blot analysis (Fig. 1B). A few were selected for PCR genotyping and confirmed to be *Mrad1 *heterozygous (Fig. 1C).

*Mrad1*^+/- ^ES cells were used to generate *Mrad1 *targeted mice (see **Methods **for details). Genotypes of the mice were analyzed by Southern blot hybridization (Fig. 1D) and PCR (Fig. 1E), and indicated that *Mrad1*^+/- ^animals were successfully generated. Mating between *Mrad1*^+/- ^mice only produced *Mrad1*^+/+ ^and *Mrad1*^+/- ^offspring, providing evidence that *Mrad1*^-/- ^causes embryonic lethality. Mating between *Mrad1*^+/- ^and *Mrad1*^+/+ ^mice generated almost equal numbers of *Mrad1*^+/- ^and *Mrad1*^+/+ ^pups (111:109), suggesting no effect of *Mrad1 *heterozygous mutation on embryonic survival. *Mrad1*^+/- ^and wild type mice were maintained and monitored up to 1.5 years of age, and heterozygotes had no overt defects compared to wild type mice.

**Figure 2:**
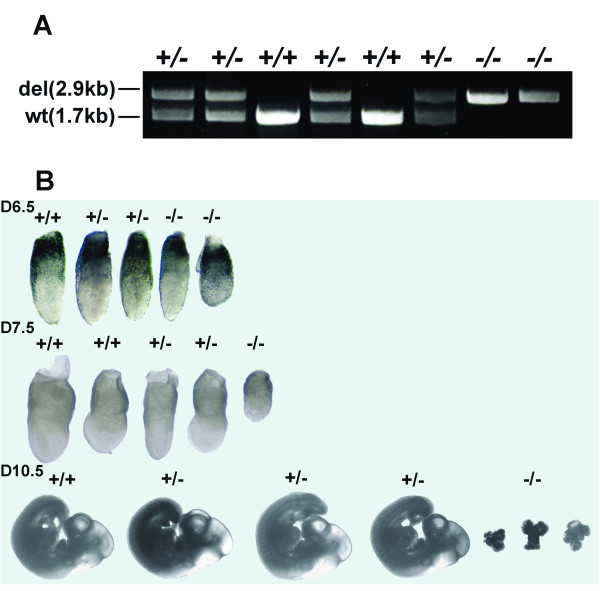
**Gross morphology of mouse embryos derived from *Mrad1*^+/- ^× *Mrad1*^+/- ^crosses**. *A*, PCR genotyping of *Mrad1 *in mice. Yolk sac genomic DNA was used as template. *B*, Representative gross morphology of *Mrad1 *mouse embryos at E6.5, E7.5 and E10.5. A complete liter of embryos at each stage are presented. +/+, *Mrad1*^+/+^; +/-, *Mrad1*^+/-^; -/-, *Mrad1*^-/-^.

Mouse embryos at 7.5 dpc with either the *Mhus1*^-/- ^or *Mrad9*^-/- ^genotype are smaller than wild-type littermates, look morphologically abnormal, and eventually die during embryonic development [21,22]. To determine the embryonic morphology and the stage of embryonic lethality of *Mrad1 *homozygous mutants, embryos resulting from *Mrad1*^+/- ^X *Mrad1*^+/- ^crosses were retrieved at different stages of gestation. We examined embryos at 6.5d, 7.5d, 8.5d, 10.5d and 11.5d. Table 1 shows the numbers and genotypes of the embryos obtained. PCR was used to genotype DNA isolated from yolk sacs (Fig. 2A). Gross morphology of embryos at the different stages of development is presented in Fig. 2B. *Mrad1*^-/-^, *Mrad1*^+/+ ^and *Mrad1*^+/- ^embryos were identified at all stages analyzed. At E6.5, 23 of the 27 embryos are close in size and morphology except 2 *Mrad1*^-/-^, 2 *Mrad1*^+/+ ^and 1 *Mrad1*^+/-^embryos were relatively smaller but with normal morphology. Overall, deletion of Mrad1 does not impact on the size and gross morphology of the embryos by E6.5. At E7.5, however, there were many *Mrad1*^-/- ^embryos significantly smaller than those with the *Mrad1*^+/- ^or *Mrad1*^+/+ ^genotype. We retrieved three liters at E7.5, and the widths of the embryos were measured. The average width among the embryos with the same genotype was calculated, and then normalized to the average width of wild type embryos. Afterwards, the average widths of the embryos from the three liters were subjected to statistical analysis (T-test), and we found that the width of *Mrad1*^-/- ^embryos was statistically significantly smaller than that of *Mrad1*^+/+ ^embryos (P = 0.016); the widths of *Mrad1*^+/- ^and *Mrad1*^+/+ ^embryos were statistically equal (P = 0.11). At E8.5, *Mrad1*^+/- ^and *Mrad1*^+/+ ^embryos were further developed while *Mrad1*^-/- ^embryos still looked like the *Mrad1*^-/- ^embryos at E7.5 (data not shown). At E10.5 only amorphous material was found as *Mrad1*^-/- ^embryos (Fig. 2B), and at E11.5 *Mrad1*^-/- ^embryos were completely resorbed. Taken together, as *Mrad9 *and *Mhus1, Mrad1 *is critical for embryonic development at or before E7.5.

**Table 1:** Numbers of embryos with indicated genotype.

Stage	*Mrad1*^+/+^	*Mrad1*^+/-^	*Mrad1*^-/-^
E6.5	8	10	9
E7.5	5	12	4
E8.5	6	17	5
E10.5	2	5	6
E11.5	5	1	3(resorptions)

### *Mrad1 *deletion enhances the incidence of mouse skin tumor development

**Figure 3:**
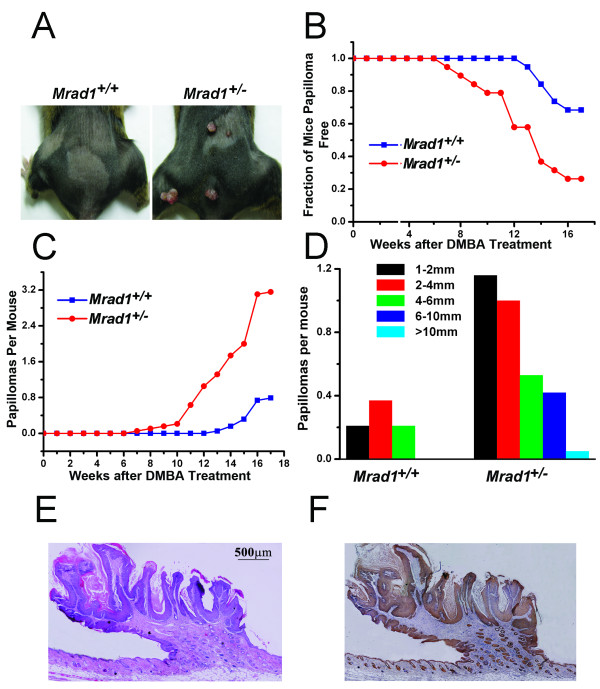
**Skin tumor induction by DMBA-TPA treatment**. *A*, Papillomas induced by DMBA-TPA treatment in *Mrad1*^+/+ ^mouse skin (left) and treated *Mrad1*^+/- ^mouse skin (right). *B*, Incidence of papilloma-free mice after DMBA-TPA treatment. Kaplan-Meier plot of tumor-free state as a function of time after DMBA painting followed by TPA treatment (blue, *Mrad1*^+/+^; red, *Mrad1*^+/-^). *Mrad1*^+/+ ^and *Mrad1*^+/- ^mice (n = 38) were initially treated once with DMBA at week 1 on the skin topically starting at ages 7 to 8 weeks, and TPA twice weekly for 17 weeks. There was a significant difference in papilloma formation between *Mrad1*^+/+ ^and *Mrad1*^+/- ^mice (P = 0.003). *C*, Average numbers of papillomas on each mouse (blue, *Mrad1*^+/+^; red, *Mrad1*^+/-^). Only papillomas larger than 1 mm diameter were counted. There was a significant difference in the number of papillomas per mouse between two the genotypes at the 17-week end point (P = 0.010). *D*, Size distribution of papillomas. The length of a papilloma was used to represent its size. *E*, H & E staining for papillomas. A typical papilloma was shown, with connective tissues extending into the tumor. *F*, Keratin 14 staining for keratinocytes. The same tumor sample in *E *was also stained for Keratin 14, and it was thus shown to be derived from keratinocytes.

To determine if *Mrad1 *is important for tumorigenesis, the skin of mice with *Mrad1*^+/+ ^and *Mrad1*^+/- ^genotypes was treated with DMBA plus TPA. A total of 38 mice were divided into 2 groups, each with the *Mrad1*^+/+ ^or *Mrad1*^+/- ^genotype. The 2 groups were from 19 litters, each litter consisting of 2 mice with *Mrad1*^+/+ ^and *Mrad1*^+/- ^genotypes, and identical sex, either female (14 liters) or male (5 liters). Under this setting, Log-Rank Test in the Kaplan-Meier PL method can be used for statistical analysis on the significance of differences of tumor development between two groups of animals [23]. DMBA was used to initiate skin tumorigenesis, and TPA was used to promote skin tumor growth. Mice were first painted with 15 μg DMBA in 100 μl acetone. One week after DMBA treatment, mice were painted with 2 μg TPA in 100 μl acetone twice a week (Monday and Thursday) for 17 weeks. Only skin tumors larger than 1 mm were recorded. After 7 weeks of TPA treatment, *Mrad1*^+/- ^mice began to develop skin tumors in the treated area. It took 13 weeks of TPA treatment for the first *Mrad1*^+/+ ^mouse to develop skin tumors (Fig. 3A and 3B). At the 17^th ^week of TPA treatment, when the experiment ended, 14 *Mrad1*^+/- ^mice had skin tumors while only 7 *Mrad1*^+/+ ^mice, treated in the same fashion, developed skin tumors (Fig. 3B). Kaplan-Meier PL method [23] was used for comparison of the relative risk of tumor development induced by DMBA plus TPA between mice with different genotypes. The rate of tumor development in *Mrad1*^+/- ^mice was significantly higher than in *Mrad1*^+/+ ^mice (P = 0.003, Log-Rank Test). Additionally, the average number of tumors in each of *Mrad1*^+/- ^mice that developed tumors was significantly higher (P = 0.010) than that in tumor-bearing *Mrad1*^+/+ ^mice (Fig. 3C). We also measured the size of tumors on mouse skin after 17 weeks of TPA treatment. Tumors larger than 6 mm in diameter only appeared in *Mrad1*^+/- ^mouse skin (Fig. 3D; data not shown). We made skew analysis on the tumor size distribution and found that G1 values were 0.40528 and 1.84488 for the wild type and *Mrad1 *heterozygous mice, respectively, suggesting that *Mrad1 *heterozygous mice bore significantly larger tumors. Staining skin specimens with H&E or anti-keratin 14 indicated that the tumors were derived from keratinocytes and possessed characteristics of papillomas (Fig. 3E and 3F). All these data suggest that Mrad1 plays an important role in the prevention of skin papilloma development.

### *Mrad1 *deletion induces spontaneous double strand DNA breaks

**Figure 4:**
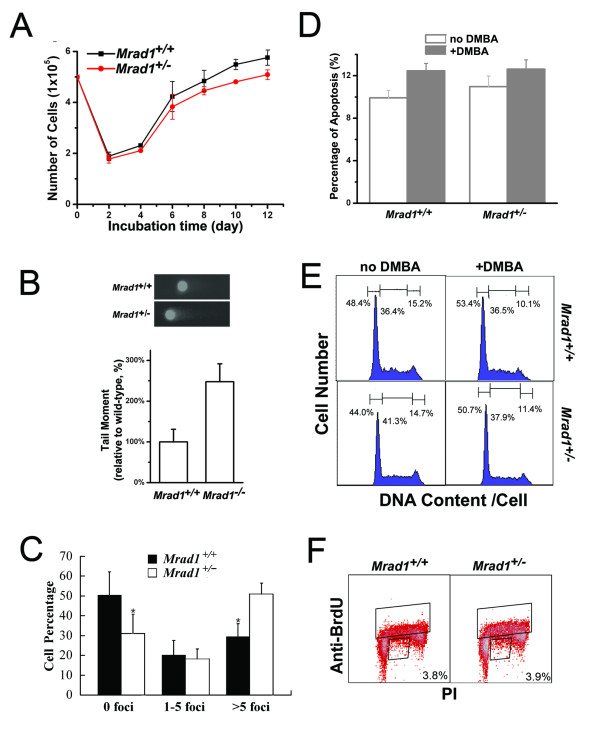
**Proliferation, spontaneous DNA DSBs, apoptosis and cell cycle distribution of *Mrad1*^+/+ ^and *Mrad1*^+/- ^keratinocytes**. *A*, Proliferation of skin keratinocytes. The average results were derived from three independent experiments. *B*, Spontaneous DNA double strand breaks detected with comet assay. The mean values were derived from three independent experiments, and each was the average of assays on 50 cells. *C*, Spontaneous DNA double strand breaks detected with γ-H2AX labeling. Assessments were made by counting foci in at least 100 cells for every sample, and the result shown is the mean of triplicate samples for each genotype. Statistical analyses: n = 3, P = 0.007 for 0 foci, P = 0.42 for 1-5 foci and P = 0.0009 for more 5 foci, *D*. Quantitative comparison of apoptosis between *Mrad1*^+/+ ^and *Mrad1*^+/- ^keratinocytes mock-treated and treated with DMBA for 24 h. The apoptotic levels were analyzed using Annexin V labeling. *E*, Comparison of cell cycle distribution of *Mrad1*^+/+ ^and *Mrad1*^+/- ^keratinocytes mock-treated and treated with DMBA. The numbers above each phase indicate the percentage of cells in that phase among the whole cell population. *Mrad1*^+/- ^cells in G1 phase were not more than *Mrad1*^+/+ ^cells in G1 phase. DMBA-TPA treatment slightly arrested cells in G1 phase. *F*, The cell cycle distributions of *Mrad1*^+/+ ^and *Mrad1*^+/- ^keratinocytes monitored with BrdUrd uptake. The cells were stained with both PI and anti-BrdUrd. The top box indicates BrdUrd-positive cells, and the number in the bottom right box is the percentage of BrdUrd-negative cells with late S phase DNA content.

DNA repair and cell cycle control are two processes important for maintaining genomic integrity and preventing carcinogenesis [1,2], thus we investigated these processes in keratinocytes bearing an Mrad1 deletion. First, we examined whether there is a significant change in the proliferation of the isolated keratinocytes resulting from the deletion of an allele of *Mrad1*. Cells were grown in defined Keratinocyte-SFM medium. Five hundred thousand cells, either *Mrad1*^+/+ ^or *Mrad1*^+/-^, were seeded into each well of 6-well plates. The number of cells in each population dropped to nearly 40% of the seeded, original number (2 × 10^5 ^per well) after the first 2 days of incubation. Subsequently, the total number of keratinocytes increased until day 12 when the experiment was terminated (Fig. 4A). There is a slight but significant difference in cell proliferation between *Mrad1*^+/+ ^and *Mrad1*^+/- ^keratinocytes (at Day 12, P = 0.03). This result indicates that *Mrad1 *deletion affects the normal proliferation of cells although the influence is not dramatic.

The isolated keratinocytes were cultured in an incubator for 3 days before analysis of DNA damage. The neutral comet assay was used to detect DNA double-strand breaks (DSBs). There were significantly more DSBs in the incubated *Mrad1*^+/- ^keratinocytes than in*Mrad1*^+/+ ^cells (P = 0.004) (Fig. 4B). The phosphorylation of histone H2AX (γ-H2AX), a marker for the presence of DSBs, was also used to evaluate DSBs in *Mrad1*^+/-^and *Mrad1*^+/+ ^keratinocytes in this study. Significantly more *Mrad1*^+/- ^keratinocytes contained γ-H2AX-positive foci than *Mrad1*^+/+ ^cells, while significantly less *Mrad1*^+/- ^keratinocytes contained no γ-H2AX foci compared to *Mrad1*^+/+ ^cells (Fig. 4C). Therefore, Mrad1 is critical for maintaining genomic integrity. We also studied the effects of Mrad1 deletion on apoptosis. The percentage of apoptotic cells among *Mrad1*^+/- ^keratinocytes is slightly higher than within the*Mrad1*^+/+ ^cell population (Fig. 4D), but the difference is not statistically significant. We also examined the apoptosis levels induced by DMBA (0.15 μg/ml, 24 h), and found that the apoptosis levels in both *Mrad1*^+/- ^and *Mrad1*^+/+ ^keratinocytes were enhanced, but there were no statistically significant differences between the DMBA-induced apoptosis levels of *Mrad1*^+/- ^and *Mrad1*^+/+ ^cells, and between the mock-treated and DMBA-treated cells with either genotypes (Fig. 4D). The above results suggest that *Mrad1 *deletion in keratinocytes does not alter DMBA-induced apoptotic response in this experimental setting.

Flow cytometric analyses of PI-stained Mrad1 keratinocytes indicated that *Mrad1*^+/- ^cells contained a slightly smaller G1 subpopulation and a slightly larger S subpopulation in the cell cycle than *Mrad1*^+/+ ^cells (Fig. 4E). After incubation for 24 h in medium containing 0.15 μg/ml DMBA, more cells with either *Mrad1 *phenotype were accumulated in G1 phase (Fig. 4E), indicating a functional G1 phase checkpoint control in both cell types. Measurement of BrdUrd uptake by replicative S phase cells in combination with DNA content via PI staining in individual cells can reveal more information on cell cycle distribution. Therefore, we investigated cell cycle profiles in more detail by pulse labeling with BrdUrd and staining cells after 4 days of incubation. There is again no major difference in cell cycle distribution between *Mrad1*^+/+ ^and *Mrad1*^+/- ^keratinocytes. The number of BrdUrd-positive *Mrad1*^+/+ ^cells in S phase is nearly the same as the number of *Mrad1*^+/- ^cells (Fig 4F). Based on the above data, we conclude that Mrad1 deletion has only a slight effect on the distribution of cell cycle phase during in vitro incubation in both mock-treated and DMBA-treated conditions. This result is consistent with the finding that *Mrad1 *deletion only leads to a slight delay in proliferation of mouse skin keratinocytes.

### Expression of the cell cycle checkpoint genes *p53*, *p21*, *Mrad9 *and *Mhus1 *in *Mrad1*^+/+ ^and *Mrad1*^+/- ^keratinocytes

**Figure 5:**
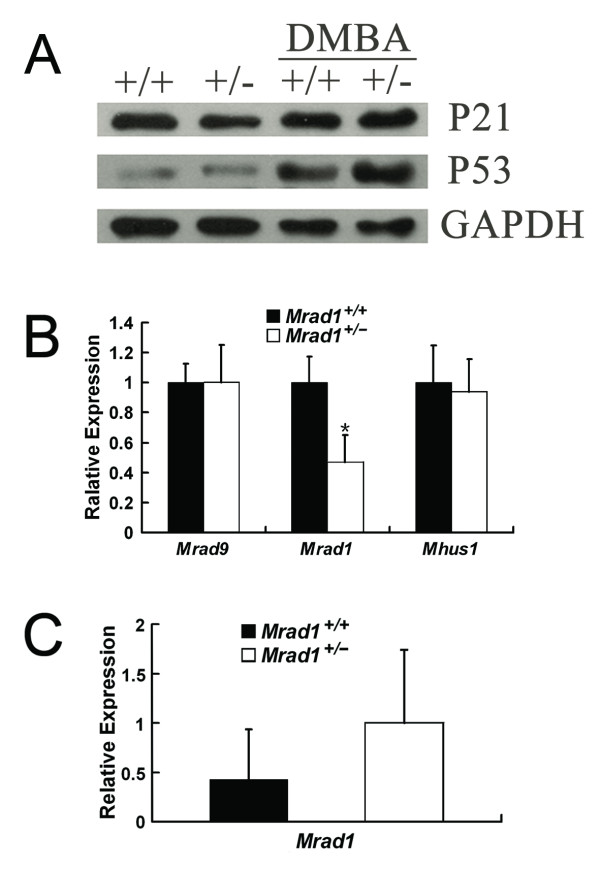
**Expression of cell cycle checkpoint genes**. A. Expression of *p21 *and *p53 *in *Mrad1*^+/+ ^and *Mrad1*^+/- ^keratinocytes. Western blotting analysis of p21 and p53 protein levels in keratinocytes incubated for four days after isolation. The first two lanes are the protein levels in cells without DMBA treatment, and the last two lanes are proteins from cells treated with 0.15 μg/ml DMBA for 24 hours. The data in the figure represent results from three independent western blotting experiments. +/+ and +/- indicate *Mrad1*^+/+ ^and *Mrad1*^+/- ^genotypes, respectively. *B*. *Mrad9 *and *Mhus1 *expression levels in *Mrad1*^+/+ ^and *Mrad1*^+/- ^keratinocytes. The gene expression levels were analyzed with real-time quantitative RT-PCR. Each result is the average ratio of the PCR results of an indicated gene relative to *β-actin *level for three independent samples, and each PCR result is the mean of triplicate PCR of the same sample. The difference of the *Mrad1 *expression levels between *Mrad1*^+/- ^and *Mrad1*^+/- ^cells is statistically significant (n = 3, P = 0.022). Either *Mrad9 *or *Mhus1 *expression levels were similar in both *Mrad1*^+/+ ^and *Mrad1*^+/- ^keratinocytes. *C*. *Mrad1 *expression levels in *Mrad1*^+/+ ^and *Mrad1*^+/- ^tumors. The expression levels of *Mrad1 *were analyzed by PCR as in *B *in this figure legend, and no statistical significance (n = 3, P = 0.34).

In a previous study, we reported that deletion of either one or two alleles of *Mrad9 *induced expression of cell cycle checkpoint genes and dramatically reduced cell proliferation of keratinocytes in culture. The results herein show that *Mrad1*^+/- ^keratinocytes have only a slightly lower proliferation rate than the *Mrad1*^+/+ ^controlcells (Fig. 4A). We therefore examined whether expression levels of *p53 *and *p21 *in *Mrad1*^+/- ^keratinocytes are higher than in the related *Mrad1*^+/+ ^cells. Indeed, heterozygous deletion of *Mrad1 *did not by itself alter expression of *p21*or *p53 *in skin keratinocytes in culture (Fig. 5A). These results can explain why *Mrad1*^+/- ^keratinocytes do not have significantly higher levels of apoptosis and only a slightly lower proliferation rate than those of the wild type keratinocyte control. We further investigated *p21 *and *p53 *expression levels in keratinocytes treated with DMBA (0.15 μg/ml, 24 h) and found that the treatment induced expression most dramatically of *p53 *but also somewhat *p21 *in both *Mrad1*^+/+ ^and *Mrad1*^+/- ^keratinocytes (Fig. 5A). The expression levels of *Mrad9 *and *Mhus1 *in keratinocytes with*Mrad1*^+/+ ^and *Mrad1*^+/- ^genotypes were also examined by real-time PCR. The results show that *Mrad9 *and *Mhus1 *expression levels are not altered by one allele *Mrad1 *deletion (Fig. 5B), suggesting that there is no regulatory effect of Mrad1 on either *Mrad9 *or *Mhus1 *expression in mouse keratinocytes. Interestingly, Mrad1 expression levels in *Mrad1*^+/- ^mouse skin tumors were higher than those in Mrad1+/^+^mouse skin tumors, but not statistically significantly (Fig. 5C).

## Discussion

Mouse *Rad1 *is homologous to *Saccharomyces cerevisiae RAD17 *(scRAD17; [24,25]), *Schizosaccharomyces pombe rad1+ (sprad1*+; [26-28]), *Ustilago maydis REC1 *[28,29] and human *Rad1 *[30-34]. In yeasts, *rad1 *is evolutionally conserved and a key component that mediates multiple cellular responses to DNA damage and cell cycle checkpoints [26,27,35-37]. After mouse and human *Rad1 *were transfected into corresponding mutant yeast cells, cell cycle checkpoint is restored. However, other functions of this gene in mammals are not well established. In this report, we examined whether *Mrad1 *prevents tumor formation and the mechanisms involved, using keratinocytes of mice with deletion of one *Mrad1 *allele because of the lethality caused by a *Mrad1 *homozygous null (Fig. 1). We showed that *Mrad1 *plays important roles in embryonic development and is required for preventing skin tumor formation induced by DMBA-TPA combinational treatment (Fig. 2 and Fig. 3). Thus we identify *Mrad1 *as an important genome caretaker or tumor suppressor for skin cancer.

*Mrad9*^-/- ^and *Mhus1*^-/- ^are critical for embryonic development at or before E7.5 [21,22]. We show in this study that homozygous deletion of *Mrad1 *also leads to embryonic lethality, and to slower growth and abnormal development at E7.5 (Fig. 2B). Therefore, the 9-1-1 (Rad9-Hus1-Rad1) complex is likely essential for normal embryonic development.

Since *Mrad1*^+/- ^mice have no readily observable abnormalities up to 1.5 years of age, we examined whether combined treatment with DMBA and TPA on skin can reveal that heterozygous deletion of *Mrad1 *causes susceptibility to tumor development. As shown in Fig. 3A, the heterozygous *Mrad1 *deletion greatly enhanced tumor development. To understand the molecular mechanism behind the tumor-preventing function of *Mrad1*, we examined cell proliferation, DNA double strand breaks, apoptosis and cell cycle phase distribution of *Mrad1*^+/+ ^and *Mrad1*^+/- ^keratinocytes. Our results indicate that heterozygous deletion of *Mrad1 *did not increase the frequency of apoptosis (minor but not statistically significant increase), the expression of *p53 *and *p21*, and only slightly reduced cell proliferation, although the *Mrad1 *deletion did induce DNA double stand breaks (Fig. 4D, A, B and 4C; Fig. 5A). In a previous study [19], we also demonstrated that heterozygous deletion of *Mrad9 *in *keratinocytes *led to spontaneous DNA double strand breaks. In addition, the heterozygous *Mrad9 *deletion induced apoptosis, high levels of *p53 *and *p21 *expression, and dramatically slowed down cell growth, which is different from what has been observed for the impact of *Mrad1 *deletion in keratinocytes. Therefore, *Mrad1 *and *Mrad9 *both prevent skin tumor development, but perhaps through different mechanisms and not exclusively via participation in the 9-1-1 complex. The 9-1-1 complex plays important roles in cell cycle checkpoint control and DNA damage repair which are important for genome stability, and thus *Mrad1 *and *Mrad9 *deletions may lead to skin tumors via loss of their genome caretaking function. Additional studies are needed to fully explain the mechanistic details and implications of these findings with respect to tumor prevention, and the impact on detection and treatment of cancer.

Among the three components in the 9-1-1 complex, human Rad9 expression has been most studied in human tumor tissues, partly due to available good anti-human Rad9 antibodies [13-16]. As mentioned above, the heterozygous deletion of *Mrad9 *induces apoptosis and dramatically reduces cell proliferation, the two features which act against tumor development and are not shared by the heterozygous deletion of *Mrad1*. Therefore, human cells with abnormal expression of human *Rad1 *or its malfunctioned mutations are more likely to survive and form tumors in patients. It would be interesting to examine Rad1 expression in human cancer tissues to find out the role of Rad1 in human tumor development.

Surprisingly, the *Mrad1 *expression level in *Mrad1*^+/- ^mice is twice that in *Mrad1*^+/+ ^mice although the difference is not statistically significant (Fig. 5C). However, the facts that the both null-*Mrad9 *and heterozygous *Mrad1 *deletion enhances susceptibility for skin tumor development, and that knockdown of highly expressed Rad9 in human prostate tumor cells correlates with reduction of tumorigenicity in nude mice [16] suggest the following models. At the very early stage, unrepaired DNA lesions enhances the opportunity for cellular genome to become more unstable and thus for the later-stage tumor development. A genome with extremely high instability does not support the cell's survival and proliferation. In the case of *Mrad9 or Mrad1 *deletion, mouse skin would become susceptible for tumor development owing to enhanced damaged cellular DNA, and in the case of human cancer, the highly expressed *Rad9 *would maintain the stability of the cancer cell genome to certain level so the cell could survive and proliferate. Obviously much more research work needs to be done to confirm the above models.

## Methods

### Targeting vector construction

A targeting vector was made to produce a deletion in *Mrad1*. We used the promoterless selection strategy to obtain a high efficiency of homologous gene targeting [20]. The targeting vector was constructed in three steps starting with pBluescript SK(+) vector. First, the 5'end fragment, a 1523 bp *Mrad1 *sequence between exon 2 and exon 3, was generated by PCR from 129 SvEv mouse genomic DNA with primers:

5'-GTCTCAGGTTTTCACACATCTTCC-3' and 5'-CTACGCGTCGACCTTCCTGAATGACAAATTCCTG-3' (Fig. 1A). The PCR product was cut with *Kpn*1 and *Sal*1, and subcloned into pBluescript SK(+). Second, the *neo *gene was amplified from pRc/CMV2 vector without the promoter and ATG using primers:

5'-CTACGCGTCGACATTGAACAAGATGGATTGCACGC-3' and 5'-AAGGAAAAAAGCGGCCGCAGACATGATAAGATACATTGATGAG-3'. Then, the PCR product was cut with *Sal*1 and *Not*1, and inserted in frame with *Mrad1 *into the plasmid constructed in the first step. Third, the 3'end fragment, 5591 bp long between intron 3 and intron 6, was generated by PCR from 129 SvEv mouse genomic DNA with primers:

5'-AAGGAAAAAAGCGGCCGCCTACTACAACTACTGCTACTAC-3' and 5'-TCCCCGCGGCACAGGACAGTACAGTAAGTCG-3'. The product was cut with *Sal*I and *Sac*II, and inserted into the vector constructed in the second step. This yielded the final targeting construct with the selectable *neo *gene, which was linearized with *Kpn*1 prior to transfection into ES cells.

### Growth of ES cells, gene targeting, and generation of *Mrad1*-deficient cells and mice

ES cells derived from 129 SvEv mice were cultured by established methods [38]. ES cells used to make gene-targeted mice were grown on feeder cells, electroporated with targeting vector linearized by *Kpn*1, and then grown in the presence of G418 at 300 μg/ml. The G418-resistant clones were picked, expanded and subjected to Southern blot hybridization and PCR analyses to identify *Mrad1*^+/- ^targeted clones. Positive clones were injected into C57BL/6 blastocysts. Chimeric offspring were born and mated to C57BL/6 mice to confirm successful germ line transmission of the targeted *Mrad1 *allele. Genomic DNA from tails was analyzed by Southern blot hybridization and PCR analyses. *Mrad1 *heterozygous mutant mice were intercrossed and maintained.

### Southern blotting and PCR assays to assess genotypes

For Southern blotting, genomic DNA was isolated from ES cells and tails of mice using published methods [22]. DNA was digested with *Hind*III, separated on a 0.7% agarose gel, then transferred to a nylon membrane, and hybridized to a ^32^P-labeled probe, which was generated by PCR using primers:

5'-GTGGCCTAGGTGGTTGCGTATCTGAAC-3' and 5'-GTCGGCTCCGAGAAGAAGGATGCTCC-3' in conjunction with mouse genomic DNA as template.

To genotype ES cells and mice by PCR, the reaction was performed using genomic DNA templates and the following primer pair:

5'-GTCTCAGGTTTTCACACATCTTCC-3' and 5'-GCTTATATTCTAGAAACCTTCCTGTATG-3'. PCR conditions were 95°C for 5 min, followed by 35 cycles of 95°C for 30 s, 59°C for 30 s, and 72°C for 3 min, with a final extension at 72°C for 10 min.

### Morphological analysis of mouse embryos

Mouse embryos were obtained at several stages of gestation, including E6.5, E7.5, E8.5, E10.5, and E11.5. All dissections were performed in 1 × PBS. Whole embryos were rinsed with 1 × PBS. Pictures of whole embryos were taken while viewed by a Wild Heerbrugg dissecting microscope.

### Preparation and in vitro culture of keratinocytes

Full-thickness skin removed from newborn (1-2 days old) mice was treated with 0.25% trypsin overnight at 4°C. The epidermis was peeled off from the dermis and minced into pieces smaller than 1 mm. They were placed into a sterile flask, then dispersed by stirring into single cells for 30-60 min, then suspended in Keratinocyte-SFM medium with supplements (Invitrogen). Cells were first incubated in dishes coated with collagen type I at 34°C in 5% CO_2 _for 12 h to allow cells to attach to the bottom. Afterwards, unattached cells were removed by washing with PBS. Attached cells were further cultured in fresh medium, which was replaced every 2 days.

### Western blotting

For preparing protein from epidermis, full-thickness skin removed from newborn mice was treated with 0.25% trypsin overnight at 4°C. The epidermis was peeled off from the dermis and dispersed in lysis buffer. To prepare cell lysate, keratinocytes incubated for 3 days were either left untreated or treated for 24 h with 0.15 μg/ml DMBA (Sigma). Then, the cell lysate was prepared in 1× SDS-sample buffer, to a final concentration of 10^4 ^cells/μL. Fifty μg of protein were resolved on a 10% SDS-PAGE gel, and proteins were transferred to a polyvinylidene difluoride membrane. The membrane was probed consecutively with primary and peroxidase-conjugated secondary antibodies. Primary and secondary antibodies used in this study are mouse anti-GAPDH (KangChen, China), mouse anti-p21 (Santa Cruz), mouse anti-p53 (Oncogene), peroxidase-conjugated anti-rabbit IgG (A9169, Sigma) and peroxidase-conjugated anti-mouse IgG (A9044, Sigma).

### DMBA-TPA induced skin tumor formation

Mice (7-8 weeks old) were shaved on their backs 2 days before tumor induction. To induce tumors, the shaved dorsal skin of mice was treated topically with 15 μg of DMBA (Sigma) in 100 μL acetone once. After 1 week, each animal received subsequent topical treatments of 2 μg of TPA (Sigma) in 100 μL acetone twice weekly for 17 weeks. Treated areas were examined weekly for the presence of tumors, which were scored positive if they reached at least 1 mm in diameter.

### Histologic analysis and Immunohistochemistry

Dorsal skin samples and tumors were fixed in 4% paraformaldehyde at 4°C overnight, embedded in paraffin, and sectioned as 8-μm slices. The sectioned tissues on slides were stained with H&E [39,40]. Immunohistochemical staining was carried out using a kit (ImmunoCruz Staining Systems, Beijing Zhongshan Golden Bridge Biotechnology). The endogenous peroxidase activity in the specimens was blocked by treatment with 0.3% H_2_O_2 _and samples were then rinsed with PBS. The specimens were probed consecutively with primary antibodies against Keratin 14 (BAbCo), secondary antibody biotin-conjugated goat anti-rabbit IgG, and horseradish peroxidase-streptavidin complex, and then visualized by diaminobenzidine. Afterwards, sections were counterstained with hematoxylin.

### Proliferation assay

Keratinocytes were isolated as described above and seeded into 6-well plates (5 × 10^5 ^cells per well) containing Keratinocyte-SFM medium with supplements. Cell numbers were determined every 2 days.

### Cell cycle analyses

The cell cycle profiles of cells in different phases were determined using previously established methods [41]. Briefely, 1 × 10^7 ^keratinocytes were plated in each 10-cm dish. After incubation for 3 days the cells were mock-treated or treated with 0.15 μg/ml DMBA (Sigma) for 24 h, then processed and stained with propidium iodide (PI), and analyzed by a FACSCalibur cytometer (Becton Dickinson). To assess DNA synthesis, 10 μM BrdUrd was added to medium and cells were pulse labeled for 40 min. Cells were then processed, probed with FITC-conjugated anti-BrdUrd antibody (Becton Dickinson) and stained with PI. Flow cytometric analyses were performed on a FACSCalibur.

### Apoptosis assay

Keratinocytes incubated for 4 days were mock-treated or treated for 24 h with 0.15 μg/ml DMBA, trypsinized for 10 min using 0.1% trypsin at 37°C, washed twice with cold PBS, then resuspended in 1× binding buffer [10 mmol/L HEPES (pH 7.4), 140 mmol/L NaCl, and 2.5 mmol/L CaCl_2_] at a concentration of 1 × 10^6 ^cells/mL. Then cells were stained with Annexin V-FITC (Jingmei Biotech) and PI for 15 min at room temperature before flow cytometric analysis.

### Neutral comet assay

Keratinocytes were cultured in standard medium for 4 days. The comet assay was carried out according to the manufacturer's instructions (Trevigen). Briefly, cells at a concentration of 1 × 10^5^/mL were mixed gently with premelted low-temperature-melting agarose at a volume ratio of 1 to 10 (v/v) and spread on glass slides. The slides were then submerged in precooled neutral lysis buffer at 4°C for 30 min. After rinsing, the slides were equilibrated in Tris-borate EDTA solution, electrophoresed at 1.0 V/cm for 20 min, and then stained with PI. Fluorescence images for at least 50 nuclei were captured using a Nikon microscope and analyzed by CASP-1.2.2 software (University of Wroclaw) for tail moment (i.e., the geometric mean of fluorescence on the tail from the nucleus).

### Statistical analysis

All statistical analyses were performed using statistical software package SPSS Version 10.0. The Kaplan-Meier PL method [23] was used for comparison of the relative risks of tumor development induced by DMBA-TPA between the mice with the two different *Mrad1* genotypes. We designed the tumor development experiment to meet a set of conditions so the Log-Rank Test in the Kaplan-Meier PL method could be used. The Student's t test was performed to determine statistical significance of the differences for the comet assay. Wilcoxon rank-sum test was used to compare the difference in tumor numbers between the two groups of mice having different *Mrad1* genotypes. In all the above analyses, a P value of < 0.05 was considered statistically significant. Skewness was used to compare the difference of tumor size distributions between *Mrad1 *wild type and heterozygous mice.

### Immunofluorescence assay

Keratinocytes grown on coverslips were fixed with 4% paraformaldehyde in PBS for 15 min at room temperature, washed in PBS twice, incubated in PBS containing 0.5% Triton-X100 for 15 min and in PBS containing 5% BSA and 0.1% Triton-X100 for 1 hr, and washed in PBS once, followed by incubation with anti-phospho-H2AX (Upstate) primary antibody (1:100 dilution) in PBS containing 5% BSA and 0.1% Triton-X100 for 1 hr at room temperature. Afterwards, the coverslips were washed two times for 5 min each in PBS and incubated with Texas Red -conjugated anti-mouse antibody (1:100 dilution in PBS containing 5% BSA and 0.1% Triton-X100) for 1 hr at room temperature. Finally, the coverslips were counterstained with DAPI (10 ng/ml). The images were captured using a fluorescence microscope.

### Quantitative real-time RT-PCR

Total RNA was isolated from mouse tumors (3 wild type and 3 *Mrad1 *heterozygous tumors) or keratinocytes cultured for 4 days using the RNeasy Mini kit, as described by the manufacturer (QIAGEN). Two μg total RNA were reverse transcribed in a 20 μL reaction volume to form cDNA using the SuperScript First-Strand Synthesis System for RT-PCR (Invitrogen). Real-time PCR was performed using the StepOnePlus system(ABI) with SYBR Green I (Takara) to label amplified DNA. A standard curve method of quantification was used to calculate the expression of target genes relative to the housekeeping gene *β-actin*. Experiments were performed thrice. The following primer pairs were used for the PCR reactions: Mrad9, 5'-GCCTCTTACTATCCACTTCG-3' and 5'-AGCCCTCATTGCCTCC-3'; *Mrad1*, 5'-GCCCTATTTCAGGTTGT-3' and 5'-TGCCCATCTTCATTTCT-3'; *Mhus1*, 5'-TCCCTGTCTTACCGTGTC-3' and 5'-CTCCCTTTAGGTTTGCTT-3'; *β*-actin, 5'-GTAAAGACCTCTATGCCAACA-3' and 5'-GGACTCATCGTACTCCTGCT-3'. We used the following PCR procedure: 94°C for 3 min, then 40 cycles of 94°C for 15 s, 55°C for 20 s, 72°C for 19 s, and a final extension at 72°C for 3 min.

## Competing interests

The authors declare that they have no competing interests.

## Authors' contributions

LH planned and performed DMBA/TPA study, the morphological analysis of mouse embryos, culture of keratinocytes in vitro, proliferation, cell cycle, apoptosis and all the statistical analysis, and prepare the draft version of the manuscript. ZH performed immunohistochemistry and western blot. YL carried out neutral comet assay. XW and KMH contributed to the isolation of mouse embryos. HH designed the study and contributed to the manuscript preparation, and specifically performed targeting vector construction, gene targeting, southern blot and PCR. HBL helped designed the experiments and revised the manuscript. All authors have read and approved the final manuscript.
